# Identification of low levels of neutral and functional genetic diversity in South African bontebok (*Damaliscus pygargus pygargus*)

**DOI:** 10.1002/ece3.10962

**Published:** 2024-03-06

**Authors:** Martin Ratanang Mogakala, Rae Marvin Smith, Caswell Mavimbela, Desiré Lee Dalton

**Affiliations:** ^1^ School of Science and Technology Sefako Makgatho Health Sciences University Medunsa South Africa; ^2^ Zoological Research South African National Biodiversity Institute Pretoria South Africa; ^3^ Department of Life and Consumer Sciences College of Agriculture and Environmental Sciences, University of South Africa Johannesburg South Africa; ^4^ School of Health and Life Sciences Teesside University Middlesbrough UK

**Keywords:** blesbok, bontebok, genetic diversity, microsatellites, toll‐like receptor 2

## Abstract

Bontebok (*Damaliscus pygargus pygargus*) and blesbok (*D. p. phillipsi*) are classified as separate sub‐species. The blesbok has a widespread distribution throughout South Africa and is listed as least concern by the International Union for Conservation of Nature (IUCN) Red List of Threatened Species. Bontebok on the other hand is endemic within the Cape Floristic Region of the Western Cape in South Africa and has been listed as near‐threatened species on the IUCN Red List of Threatened Species. Bontebok populations experienced a severe bottleneck and were brought back from the brink of extinction in the 1830s. Currently, the subspecies is threatened by hybridisation with blesbok resulting in fertile offspring. To date, molecular investigations using neutral markers have determined that genetic diversity in pure South African bontebok was significantly lower than in pure blesbok. Here, we investigated genetic diversity in bontebok, blesbok and hybrid individuals using microsatellites and an adaptive marker (toll‐like receptor two (TLR2)). The study of single nucleotide polymorphisms (SNPs) revealed five mutations in TLR2 in different individuals and subspecies of *D. pygargus*. This included three non‐synonymous and two synonymous mutations. The three amino acid substitution mutations were predicted to have no effect on protein function. Two of the five mutations, one of which resulted in an amino acid substitution, were not present in bontebok. The other three mutations were present to varying frequencies in the three groups. We confirm low adaptive and neutral diversity in bontebok. These mutations provide insights into the genetic diversity and relationships among the two sub‐species of *D. pygargus* and may have implications for their conservation and management.

## Graphical abstract

Assessment for the first time, patterns of neutral and immunity‐related genetic diversity in pure and hybrid animals. Amino acid substitutions are all tolerated, which may be attributed to purifying selection. We confirm low adaptive and neutral diversity in bontebok.

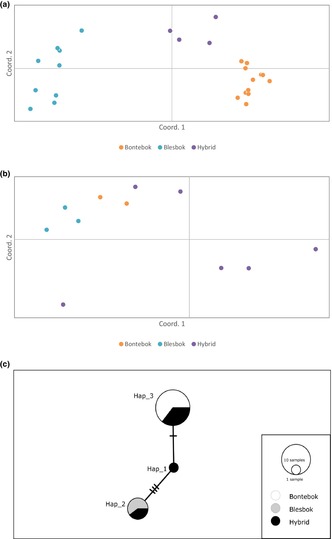

Bontebok (*Damaliscus pygargus pygargus*) and blesbok (*D. p. phillipsi*) have been classified as separated sub‐species (van der Walt et al., 2013; van Wyk et al., 2013). The blesbok have a widespread distribution in South Africa (Skinner & Smithers, 1990) with an estimated population size of 235,000–240,000 (East, 1999). Bontebok are endemic to the Cape Floristic Region of the Western Cape in South Africa (Radloff et al., 2017; Figure 1). In the early 1800s, the bontebok populations experienced a severe bottleneck due to overhunting and disease (van Rensburg, 1975), and the current number of bontebok is estimated between 6500 and 7000 animals (Birss et al., 2013). Bontebok are currently threatened by low habitat availability, hybridisation with blesbok and low genetic diversity (van Wyk et al., 2013). In addition, disease may lead to further population declines with an outbreak of an epizootic disease being identified as having detrimental consequences to bontebok populations (Skead, 1964). For example, copper deficiency, massive worm infestations and related syndromes have been reported to cause large mortalities in bontebok (50%; Barnard & Van der, 1961). Due to selective forces, the effects on genetic diversity may differ between neutral loci such as microsatellites and between adaptive loci such as the major histocompatibility complex (MHC) which is essential for the adaptive immune system and toll‐like receptors (TLRs) which plays a key role in the innate immune system (Knafler et al., 2017). An analysis of MHC class II locus DRB has identified a higher level of polymorphism in the blesbok (22 alleles) than in the bontebok (6 alleles) which was attributed to bottleneck events in bontebok (Van der Walt et al., 2001). Here, we expand on that study to investigate the level of genetic diversity at adaptive (TLR) and neutral sites in bontebok, blesbok and hybrid individuals. TLR2 was selected as it exhibits high levels of polymorphism in several mammalian species (Bartens et al., 2021) and recognises the largest number of pathogen‐associated molecular patterns (PAMPs), detecting components from bacteria, viruses and fungi (Barbalat et al., 2009).

**FIGURE 1:**
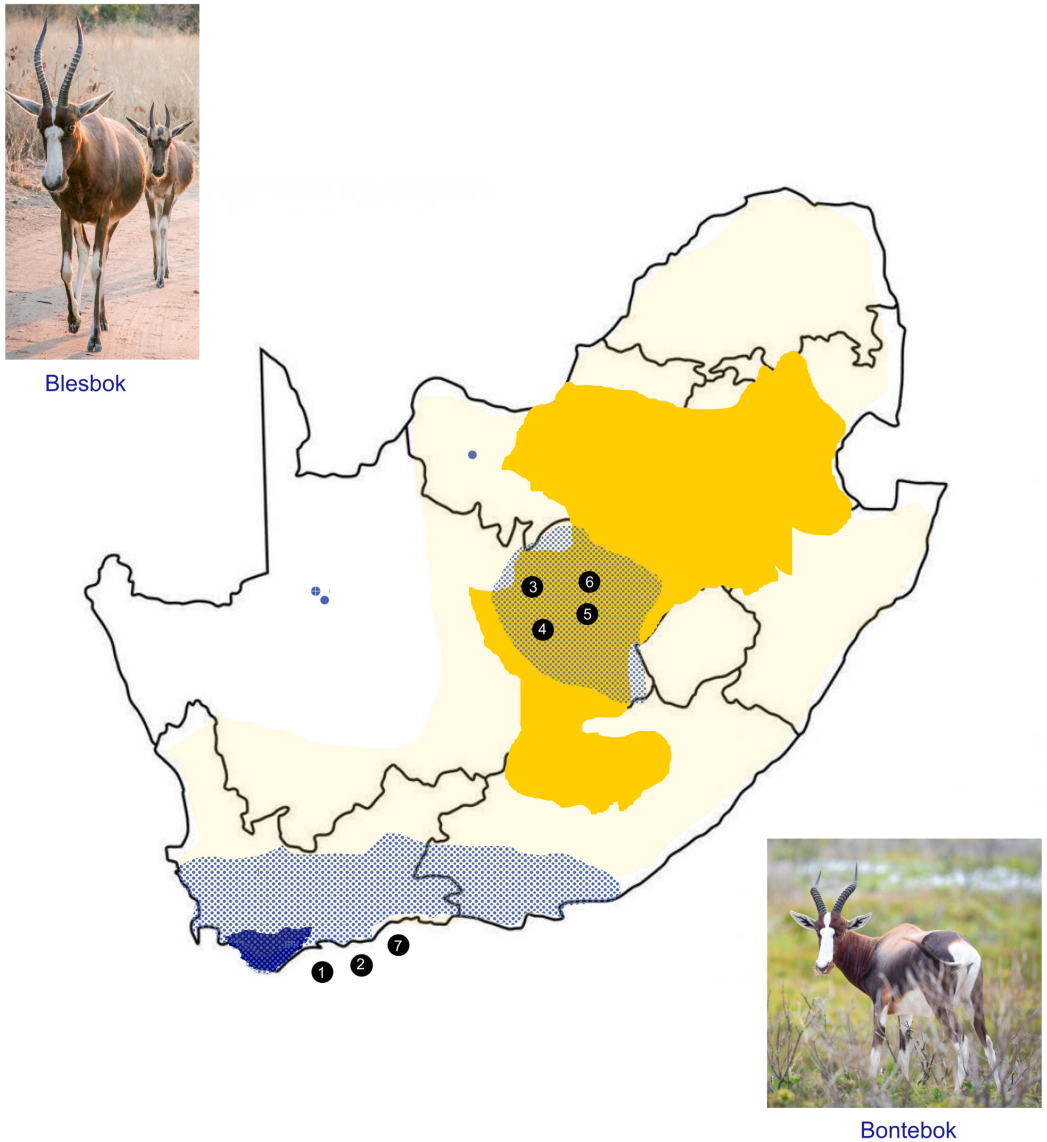
Historical and current distribution ranges of the bontebok (historically indicated as solid blue, current indicated as blue dots) and blesbok (historically indicated as solid dark yellow, current indicated as solid light yellow). Black lines indicate the South African boundary. Pure bontebok samples were collected from the Western Cape (numbers 1 and 2) and the Free State (number 3), pure blesbok were collected from the Free State (numbers 4 and 5), and hybrids were collected from the Free State (number 6) and the Western Cape (number 7).

Biological materials were collected from eight populations in the Western Cape and Free State provinces of South Africa and included 20 bontebok, 15 blesbok and 15 hybrid individuals. Tissue and blood samples were provided by the South African National Biodiversity Institute (SANBI) Biobank. DNA extraction was conducted using the ZR Genomic DNA™‐Tissue Miniprep kit (Zymo Research), according to the manufacturer's protocol. A total of 13 microsatellites were amplified as described by van Wyk et al. (2013). These markers have been validated to distinguish between pure and hybrid animals. In summary, amplification was carried out in 15 μL reactions which consisted of 1× buffer, 2.5 mM MgCl_2_, 200 mM of each 2′‐deoxynucleotide triphosphate (dNTP), 10 pico mol (pmol) of each of the forward and reverse primer, 1‐unit (U) Taq DNA polymerase and 10–20 nanogram (ng) genomic DNA template. The conditions for PCR amplification were as follows; 5 min (min) at 95°C initial denaturation, 30 cycles for 30 s (sec) at 95°C, 30 s at 50–65°C and 30 s at 72°C, followed by extension at 72°C for 20 min. PCR products were pooled together and run against GeneScan™ 500 LIZ™ (Applied Biosystems, Inc.) internal size standard on an ABI 3130 Genetic Analyzer (Applied Biosystems). Samples were genotyped using GeneMapper v. 4.0. Three primer sets of TLR2 were designed based on a reference sequence of blesbok (EU580541.1) to amplify the entire exon of TLR2 (Table S1). Amplification was carried out using 1× DreamTaq Green PCR Master Mix, following the manufacturer's protocol. The temperature profile was as follows: denaturation at 95°C for 3 min, 35 cycles of 95°C for 30 s, 55–65°C for 30 s, and 72°C for 30 s, followed by a final extension at 72°C for 10 min. Gene fragments were sequenced in both directions using the BigDye™ Terminator v3.1 Cycle Sequencing Kit, purified using the BigDye XTerminator™ Purification Kit (Applied Biosystems), and were visualised on a 3500 Genetic Analyzer. Sequence chromatograms were edited and assembled using Geneious v. R10.2 (Biomatters inc). All sequences have been deposited in GenBank (Accession nos OQ774780–OQ774808). Single nucleotide polymorphisms (SNPs) were identified manually by the identification of double peaks in Geneious v10.2.6. Synonymous and non‐synonymous SNP variations were determined by translating the TLR gene nucleotide sequences to the longest open reading frames. The identity and integrity of the respective amino acid sequences were confirmed by standard protein Basic Local Alignment Search Tool (BLAST). MICRO‐CHECKER was used (Van Oosterhout et al., 2004) to detect potential genotyping errors and null alleles for microsatellite data. Linkage disequilibrium (LD) and deviations from Hardy–Weinberg equilibrium (HWE) were calculated using ARLEQUIN version 3.5.1.2 (Excoffier & Lischer, 2010). Genetic diversity within populations was determined using GenAlEx version 6.5 (Peakall & Smouse, 2012). Genetic differentiation among subspecies was evaluated using principal component analysis (PCA) in GenAlEx 6.5. A median‐joining network haplotype map (Bandelt et al., 1999) was created for 20 of the samples no missing data using PopArt v1.7 (Leigh & Bryant, 2015, https://popart.maths.otago.ac.nz/). The amino acid domains were predicted using the online utility ExPASy PROSITE (http://prosite.expasy.org, Sigrist et al., 2012) by running the *D. d. phillipsi* TLR2 reference gene. Sorting Intolerant from Tolerant (SIFT) algorithm (https://sift.bii.a‐star.edu.sg/) was used to determine if the non‐synonymous mutations are tolerated or intolerant relative to adjacent positions, following the SIFT algorithm. A three‐dimensional structure of the TLR2 protein (UniProtKB: B2LT63) previously modelled using AlphaFold was obtained (Mirdita et al., 2022). Positions where amino acid mutations were present were modified to visualise spatial positioning within the secondary and tertiary protein structure.

The panel of 13 microsatellite markers was obtained in 19 bontebok, 11 blesbok and 11 hybrids. DNA sequences of TLR2 (~1783 bp) were successfully amplified in 14 bontebok, 11 blesbok and 11 hybrid individuals. Lack of amplification in some samples was attributed to DNA degradation. Linkage disequilibrium, null alleles and deviations from HWE were not detected in the microsatellite data or TLR2 SNP data. The PCA analysis revealed clear structuring of subspecies based on microsatellite data (Figure 2a); however, analysis of adaptive markers revealed weak structure (Figure 2b). The Haplotype map (Figure 2c) shows three haplotypes for which the bontebok and blesbok sub‐species group separately while hybrid animals feature within each. A single hybrid presented heterozygous bases in three positions resulting in the intermediate cluster, Haplotype 1. Five mutations (C132T, T140C, A223G, C463G, T1563C) were identified in TLR2 in different individuals and subspecies of *D. pygargus*. This comprised three synonymous mutations and two non‐synonymous mutations (Table 1; Table S2). The three mutations (T140C, A223G and C463G) that resulted in amino acid substitutions (F47S, M75V and L155V) which were predicted to not affect protein function. Three of the five mutations (C132T, T140C and T1563C) were not present in bontebok, one of which resulted in an amino acid substitution. The other two mutations (A223G and C463G) were present in the three groups at varying frequencies. Analysis of genetic diversity at neutral loci indicated higher diversity in blesbok samples (*H*_o_ = 0.36 ± 0.068) compared to bontebok samples (*H*_o_ = 0.22 ± 0.073) (Table 2) with hybrid individuals displaying similar diversity to bontebok (*H*_o_ = 0.22 ± 0.070). At adaptive loci, hybrid individuals had the highest diversity (*H*_o_ = 0.126 ± 0.019), followed by blesbok (*H*_o_ = 0.0683 ± 0.083) and bontebok (*H*_o_ = 0.047 ± 0.029). Here, we used a SIFT program to predict whether an amino acid substitution affects protein function, to prioritise which substitutions to investigate further. Results of SIFT predictions (Figure S1) demonstrated that the amino acid substitution on modified sites was all tolerated and do not affect protein function and thus do not alter phenotype.

**FIGURE 2:**
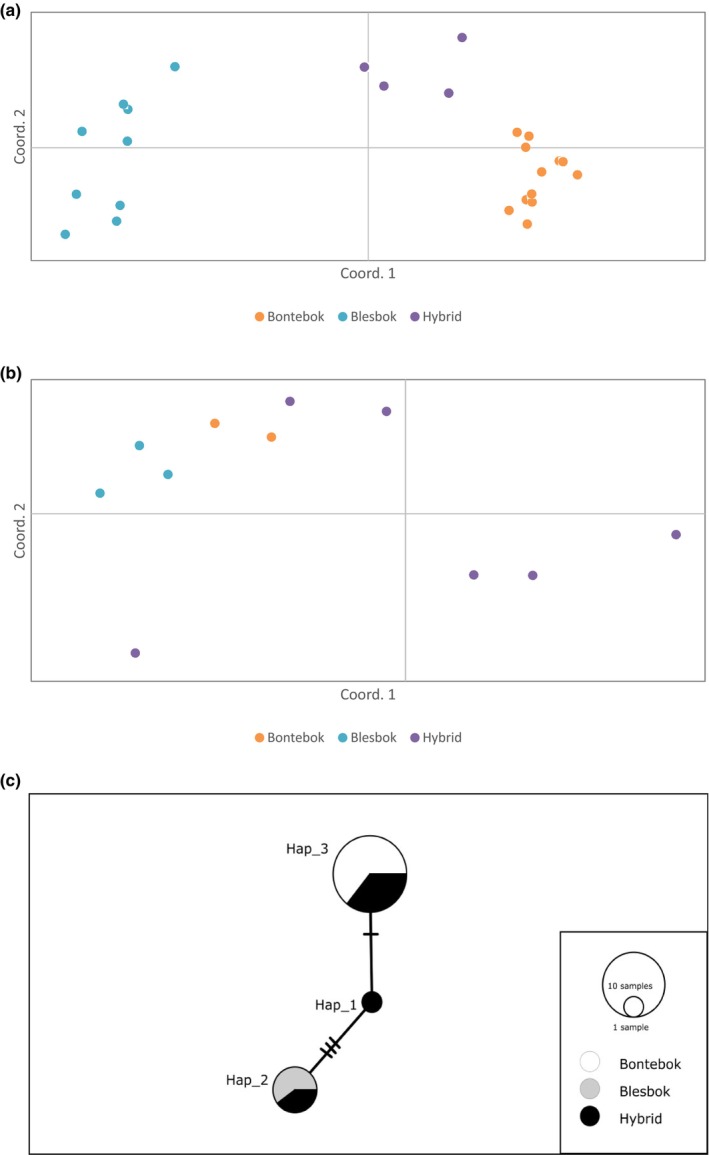
Principal coordinate analysis (PCA) of bontebok, blesbok and hybrid populations based on (a) microsatellite analysis and (b) toll‐like receptor 2 data. For microsatellite data, the first two axes explained 68.12% of the variation (the first axis explained 50.42%, the second axis, 17.7%). For toll‐like receptor 2 data, the first two axes explained 86.15% of the variation (the first axis explained 53.62%, the second axis, 32.53%). (c) Haplotype network of TLR data, spanning three haplotypes that represent 20 samples that had no missing data.

**TABLE 1:** Single nucleotide polymorphisms (SNPs) detected in the toll‐like receptor 2 gene of bontebok, blesbok and hybrid individuals from eight populations.

aa Position	S44S		F47S		M75V		L155V		F521F	
DNA position	C132T		T140C		A223G		C463G		T1563C	
Western Cape Population 1	75%	CT	75%	TC	75%	AG	100%	CG	25%	TC
	0%	GC	0%	TT	0%	AA	0%	CC	0%	TT
	0%	TT	0%	CC	0%	GG	0%	GG	0%	CC
	25%	Nd	25%	Nd	25%	Nd	0%	Nd	75%	Nd
Western Cape Population 2	0%	CT	0%	TC	0%	AG	0%	CG	0%	TC
	0%	GC	0%	TT	0%	AA	0%	CC	0%	TT
	0%	TT	0%	CC	0%	GG	0%	GG	0%	CC
	100%	Nd	100%	Nd	100%	Nd	100%	Nd	100%	Nd
Free State Population 3	40%	CT	40%	TC	40%	AG	73%	CG	47%	TC
	0%	GC	0%	TT	0%	AA	7%	CC	7%	TT
	0%	TT	0%	CC	0%	GG	0%	GG	0%	CC
	60%	Nd	60%	Nd	60%	Nd	20%	Nd	46%	Nd
Free State Population 4	0%	CT	0%	TC	63%	AG	63%	CG	0%	TC
	13%	GC	13%	TT	13%	AA	13%	CC	0%	TT
	0%	TT	0%	CC	0%	GG	0%	GG	0%	CC
	87%	Nd	87%	Nd	24%	Nd	24%	Nd	100%	Nd
Free State Population 5	0%	CT	0%	TC	0%	AG	0%	CG	0%	TC
	0%	GC	0%	TT	0%	AA	0%	CC	0%	TT
	0%	TT	0%	CC	0%	GG	0%	GG	0%	CC
	100%	Nd	100%	Nd	100%	Nd	100%	Nd	100%	Nd
Free State Population 6	27%	CT	55%	TC	88%	AG	53%	CG	0%	TC
	18%	GC	18%	TT	12%	AA	12%	CC	0%	TT
	0%	TT	0%	CC	0%	GG	0%	GG	0%	CC
	55%	Nd	27%	Nd	0%	Nd	35%	Nd	100%	Nd
Western Cape Population 7	100%	CT	100%	TC	100%	AG	100%	CG	0%	TC
	0%	GC	0%	TT	0%	AA	0%	CC	0%	TT
	0%	TT	0%	CC	0%	GG	0%	GG	0%	CC
	0%	Nd	0%	Nd	0%	Nd	0%	Nd	100%	Nd
Unknown Population 8	80%	CT	20%	TC	0%	AG	20%	CG	0%	TC
	20%	GC	40%	TT	100%	AA	60%	CC	60%	TT
	0%	TT	0%	CC	0%	GG	0%	GG	0%	CC
	0%	Nd	40%	Nd	0%	Nd	20%	Nd	40%	Nd

*Note*: The amino acid (aa) residue, the position and the respective DNA position are shown. Non‐synonymous amino acid (aa) residue, the position and respective DNA positions are shown below.

Abbreviation: Nd, no data.

**TABLE 2:** Genetic diversity estimates for bontebok, blesbok and hybrids based on (A) microsatellite data and (B) Single nucleotide polymorphism (SNP) data from toll‐like receptor 2 (TLR2).

Population	*N*_a_	*N*_e_	*H*_o_	*H*_e_	*uH*_e_
A. Microsatellite data					
Bontebok	1.769 (0.201)	1.425 (0.155)	0.224 (0.068)	0.215 (0.065)	0.229 (0.070)
Blesbok	2.615 (0.290)	2.030 (0.249)	0.360 (0.073)	0.408 (0.072)	0.433 (0.077)
Hybrid	1.923 (0.329)	1.406 (0.191)	0.215 (0.070)	0.243 (0.068)	0.270 (0.076)
B. TLR2 SNP data					
Bontebok	1.500 (0.289)	1.050 (0.030)	0.047 (0.029)	0.045 (0.027)	0.046 (0.028)
Blesbok	1.250 (0.250)	1.096 (0.096)	0.083 (0.083)	0.069 (0.069)	0.074 (0.074)
Hybrid	2.000 (0.000)	1.649 (0.017)	0.126 (0.019)	0.394 (0.006)	0.415 (0.007)

*Note*: Standard error (SE) is indicated in brackets.

Abbreviations: *H*_e_, expected heterozygosity; *H*_o_, observed heterozygosity; *N*_a_, mean number of alleles per locus; *N*_e_, mean number of effective alleles per locus; *uH*_e_, unbiased expected heterozygosity.

In this study, we assessed for the first time patterns of neutral and immunity‐related genetic diversity in pure and hybrid animals and characterised the SNP mutations found. Molecular characterisation and SIFT results indicated that the amino acid substitution on modified sites were all tolerated which may be attributed to purifying selection which operates to preserve the products of most protein‐coding genes (Hughes et al., 2003). Microsatellite data identified clear structuring of the subspecies and hybrids whereas immunity loci showed clear cluster differentiation of bontebok and blesbok but not hybrids in the PCA. This observation may be attributed to the higher mutation rate of microsatellite markers in comparison to SNPs. In addition, a larger number of SNPs is required to provide the same power to detect population structure as microsatellites (Pritchard & Rosenberg, 1999; Rosenberg et al., 2003). Analysis of genetic diversity based on TLR2 analysis indicated higher diversity in hybrid individuals which was not reflected based on microsatellite data. In general, bontebok and blesbok were homozygous for SNPs, whereas hybrids were heterozygous due to the mixing of the two subspecies, which resulted in a gain of diversity. In contrast, bontebok, blesbok and hybrids were generally heterozygous at several loci with specific alleles being private for each subspecies. Thus, in this case, hybrid vigour is due to heterozygote advantage, which will be rapidly lost in subsequent generations due to segregation of gametes (Hansson & Westerberg, 2002). However, further analysis of the fitness consequences of hybridisation between bontebok and blesbok is advocated. Here, we identified lower genetic diversity in the bontebok compared to blesbok at both neutral and adaptive loci which may be attributed to the genetic bottleneck described in the 1800s (van Wyk et al., 2013). Low adaptive genetic diversity for bontebok has been previously reported for the MHC class II locus DRB (Van der Walt et al., 2001). Although it is difficult to determine the consequences of low genetic diversity to species viability, these results suggest that bontebok may have a limited ability to adapt to environmental changes and may be unable to mount appropriate immune responses against novel pathogens. Although bontebok populations have expanded in recent years, they still face several threats including habitat loss, hybridisation with blesbok and being kept in small populations. It is thus recommended that residual genetic variation be maintained through managed breeding and translocations between reserves. Further studies are recommended: (1) genomic approaches should be used to assess patterns of genomic erosion that might limit their viability (2) reproduction and survival rates should be calculated by determining the proportion of abnormal sperm and by monitoring number of surviving offspring in a population and (3) due to the threats of stochastic events such as disease, population should be monitored by regular disease/pathogen screening.

## AUTHOR CONTRIBUTIONS

**Martin Ratanang Mogakala:** Data curation (equal); formal analysis (equal); investigation (equal); methodology (equal); writing – original draft (equal). **Rae Marvin Smith:** Data curation (equal); formal analysis (equal); methodology (equal); software (equal); supervision (equal); writing – review and editing (equal). **Caswell Mavimbela:** Supervision (equal); writing – review and editing (equal). **Desiré Lee Dalton:** Conceptualization (lead); methodology (equal); project administration (lead); supervision (equal); writing – review and editing (equal).

## CONFLICT OF INTEREST STATEMENT

The authors declare no conflicts of interest.

## Supporting information


Figure S1.

Table S1.

Table S2.

## Data Availability

All sequences have been deposited in GenBank (Accession nos OQ774780–OQ774808).
