# Supplementary figures and images for: Identification of low levels of neutral and functional genetic diversity in South African bontebok (Damaliscus pygargus pygargus)
Source: Ecol Evol. 2024 Mar 6;14(3):e10962. doi: 10.1002/ece3.10962 (PMC10915478; doi:10.1002/ece3.10962)

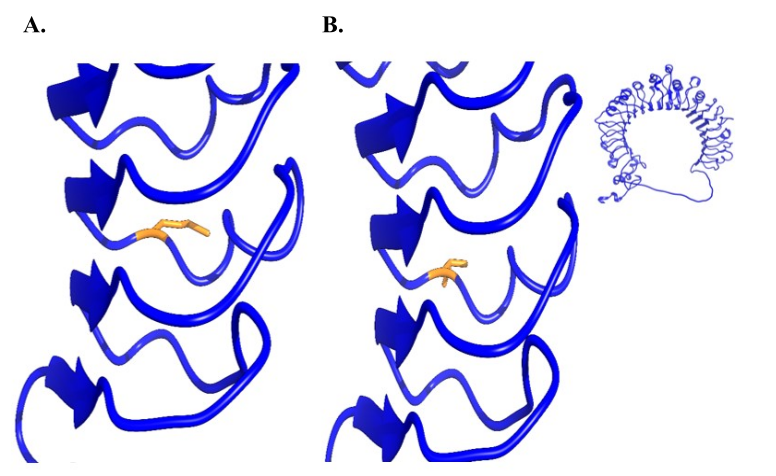

Supplement: Supplementary file 1 — Figure S1. [file ECE3-14-e10962-s002.png]
