# Supplementary material for: Identification of low levels of neutral and functional genetic diversity in South African bontebok (Damaliscus pygargus pygargus)
Source: Ecol Evol. 2024 Mar 6;14(3):e10962. doi: 10.1002/ece3.10962 (PMC10915478; doi:10.1002/ece3.10962)
Supplement: Supplementary file 2 — Table S1. [file ECE3-14-e10962-s003.docx]

**Supplementary material**

**Supplementary Table 1:** List of primers designed and used in this study.

| **Primer name** | **Sequences** | **Length (bp)** |
| --- | --- | --- |
| TLR2_F1_F | 5’-CACATGCTTTGTGGACAGC-3’ | 19 |
| TLR2_F1_R | 5’-TCGCCAATTCCATCATGGGTAC-3’ | 22 |
| TLR2_F2_F | 5’-GAAATGTGCAATTCACCGATGA-3’ | 22 |
| TLR2_F2_R | 5’-AGGAAGTCACAGGAGCAA-3’ | 18 |
| TLR2_F3_F | 5’-ACTCTACCAGATGCCTCCT-3’ | 19 |
| TLR2_F3_R | 5’-TAGGACCTTATTGCAGCTCTC-3’ | 21 |
