# Supplementary material for: Identification of low levels of neutral and functional genetic diversity in South African bontebok (Damaliscus pygargus pygargus)
Source: Ecol Evol. 2024 Mar 6;14(3):e10962. doi: 10.1002/ece3.10962 (PMC10915478; doi:10.1002/ece3.10962)
Supplement: Supplementary file 3 — Table S2. [file ECE3-14-e10962-s001.docx]

**Supplementary Table 2:** Blesbok (*Damaliscus pygargus phillipsi*) toll-like receptor 2 (TLR2) gene control DNA sequence (EU580541.1) obtained from the National Center for Biotechnology Information (NCBI). Primer set 1 (forward and reverse primers) are indicated in underlined bold blue text, primer set 2 is indicated in underlined bold orange text and primer set 3 is indicated in underlined bold gray text. The Single nucleotide polymorphism sites are indicated in bold black and underlined.

| ATGC**CACATGCTTTGTGGACAGC**GTGGGTCTGGGCTGTAATCAGCGTGTCCACGGAAGGAGCCTCTGTGGCTTCTTCTCTGTCTTGTGACCCAACTGGTGTCTGCGATGGCCATTCCAGATCTTTAAACTC**Y**ATCCCCT**Y**TGGTCTCACGGAAGGTGTGAAAAGCCTTGACCTGTCCAACAACGAGATCACCTATGTCAGCAACAGAGACCTGCAGAGGTGT**R**TGAACCTGAAGACTCTGAGGCTGGGGGGCAATGAAATTCACACGGTGGAGGAAGATTCTTTTTTTCACCTGAGGAATCTTGAATATTTGGACTTATCCTATAATCGCTTATCTAACTTATCATCCTCCTGGTTCAGGTCCCTTTATGTCTTAAAATTCTTAAACTTACTGGGAAATTTATACAAAACACTTGGGGAAACATCCCTTTTTTCTCATCTCCCAAATCTGCGGACCCTGAAA**S**TAGGAAATAGTAACAGCTTCACTCAGATTCATGAAAAGGATTTCACTGGACTGACTTTTCTTGAGGAGCTTGAGATCAGTGCTCAAAATYTGCAGATATATGCGCCAAAGAGTTTAAAGTCAATCCACAACATTAGCCATCTGATTCTTCATCTGAAGCAGCCTGTTTTACTCCTGG ATCCCCTACATTCTTGTAGATATTGTAAGTTCCTTAGATTATTTAGAACTGAGAGATACTAATTTGCACACTTTCTATTTTTCAGAAGCATCCATCAGTGAAATTAATACATCAGTTAAAAAGCTTATATTTA**GAAATGTGCAATTCACCGATGA**AAGTTTTGTTGAAGTTGTCAAACTGTTTAACTATGTTTCTGGGATCTTAGAAGTAGAGTTTGATGACT**GTACCCATGATGGAATTGGCGA**TTTTAGAGCACTGACTTTTAACAGAATTAGATACCTAGGTAACGTGGAGACGTTAACAATACGGAAGTTGCATATCCCACAGTTTTTCTTATTTCATGATCTGAGTAGTATATATCCACTCACAGGTAAAGTTAAAAGAGTCACAATAGAAAACAGTAAGGTTTTCCTGGTTCCTTGTTTACTTTCACAACATTTAATATCGCTAGAATATTTGGATCTCAGTGAAAACTTAATGTCTGAAGAAACCTTGAAAAACTCAGCCTGTGAGCATGCCTGGCCCTTCCTTCAAACCCTGGTTTTAAGGCAGAATCGTTTGAAATCACTAGAAAAAACTGGAGAACTTTTGCTTACTCTGAAAAATCTGAATAACCTTGATATCAGTAAGAATAATTTTCTTTCAATGCCTGAAACTTGTCAGTGGCCAGGAAAAATGAAACAGTTGAACTTATCCAGCACGAGGATACACAGTTTAACCCAGTGCCTTCCCCAGACCCTGGAAATTTTAGATGTTAGCAATAACAATCTCGATTCATTTTCTTTGATTTTGCCGCAACTCAAAGAACTGTATATTTCCAGAAATAAGTTGAAG**ACTCTACCAGATGCCTCCT**TCTTACCCGTGTTATCAGTTATGAGAATTAGCGGAAATATAATAAATACTTTCTCGAAGGAACAACTTGATTCTTT**S**CCACAACTGAAGGCTTTGGAGGCCGGTGGCAACAACTTCAT**TTGCTCCTGTGACTTCCT**GTCCTTCACACAGGGACAGCAAGCACTGGCCCGTGTCCTGGTCGACTGGCCAGATGGCTACCGCTGTGACGCTCCCTCGCACGTGCGGGGCCAGCGGGTGCAGGACGCCCGGCTCTCCCTTTCTGAATGCCACCGGGCGGCCGTGGTGTCTGCCGTGTGCTGTGCCCTTTTCCTGTTGCTCCTGCTCACGGGGGCGCTGTGTCACCGTTTCCACGGGCTGTGGTACATGAAGATGATGTGGGCCTGGCTCCAGGCCAAGAGGAAGCCCAGGAAGGCTCCCCGCAGGGACCTCTGCTACGACGCCTTTGTGTCCTACAGTGAGCGGGATTCCTACTGGGTGGAGAACCTCATGGTCCAGGAGCTGGAGCACTTCAACCCCCCCTTTAAGCTGTGTCTTCATAAGCGAGACTTCGTTCCTGGCAAGTGGATTATCGACAACATCATTGACTCCATTGAGAAGAGCCACAAAACCATCTTTGTGCTTTCGGAGAACTTTGTGAAGAGCGAGTGGTGCAAGTATGAGCTGGACTTCTCCCATTTCCGTCTCTTTGATGAGAACAATGATGCTGCCATTCTGATTCTGCTGGAGCCCATTGACAAGAAGGCCATTCCCCAGCGCTTCTGTAAGCTGCGGAAGATCATGAACACCAAGACCTACCTGGAGTGGCCCACGGATGAGACTCAGCAGGAAGCGTTTTGGTTAAATTT**GAGAGCTGCAATAAGGTCCTA**G |
| --- |
